# Subthalamic stimulation modulates working memory-related cortical dynamics in Parkinson’s disease

**DOI:** 10.1093/braincomms/fcag328

**Published:** 2026-08-31

**Authors:** Marius Keute, Tianlu Wang, Silvana Miranda Montenegro, Maximilian Scherer, Patrick Bookjans, Bastian Brunnett, Idil Cebi, Luka Milosevic, Daniel Weiss, Alireza Gharabaghi

**Affiliations:** Institute for Neuromodulation and Neurotechnology, University Hospital and University of Tübingen, Tübingen 72076, Germany; Institute for Neuromodulation and Neurotechnology, University Hospital and University of Tübingen, Tübingen 72076, Germany; Institute for Neuromodulation and Neurotechnology, University Hospital and University of Tübingen, Tübingen 72076, Germany; Institute for Neuromodulation and Neurotechnology, University Hospital and University of Tübingen, Tübingen 72076, Germany; Institute for Neuromodulation and Neurotechnology, University Hospital and University of Tübingen, Tübingen 72076, Germany; Institute for Neuromodulation and Neurotechnology, University Hospital and University of Tübingen, Tübingen 72076, Germany; Institute for Neuromodulation and Neurotechnology, University Hospital and University of Tübingen, Tübingen 72076, Germany; Center for Neurology, Department for Neurodegenerative Diseases, and Hertie Institute for Clinical Brain Research, University of Tübingen, Tübingen 72076, Germany; Institute for Neuromodulation and Neurotechnology, University Hospital and University of Tübingen, Tübingen 72076, Germany; Clinical and Computational Neuroscience, Krembil Research Institute, University Health Network, Toronto M5T 2S8, Canada; Max Planck-University of Toronto Centre for Neural Science & Technology (MPUTC), Toronto M5T 2S8 (Canada)/Tübingen 72076, Germany; Center for Neurology, Department for Neurodegenerative Diseases, and Hertie Institute for Clinical Brain Research, University of Tübingen, Tübingen 72076, Germany; Institute for Neuromodulation and Neurotechnology, University Hospital and University of Tübingen, Tübingen 72076, Germany; Max Planck-University of Toronto Centre for Neural Science & Technology (MPUTC), Toronto M5T 2S8 (Canada)/Tübingen 72076, Germany; Center for Digital Health (CDH), Tübingen 72076, Germany; Cognitive Science Center (CSC), Tübingen 72076, Germany; Center for Bionic Intelligence Tübingen Stuttgart (BITS), Tübingen 72076, Germany; German Center for Mental Health (DZPG), Tübingen 72076, Germany

**Keywords:** deep brain stimulation, working memory, electroencephalography, Parkinson’s disease, subthalamic nucleus

## Abstract

The dynamic modulation of large-scale network activity, which is inherent to cognitive processes, is disrupted in Parkinson’s disease. Subthalamic deep brain stimulation can either improve or deteriorate cognition, particularly executive function, with these effects often going unnoticed during acute parameter optimization. This highlights the need for longer stimulation periods and more focused research on the underlying cortical mechanisms, which remain underexplored. This study was a prospective clinical trial involving nineteen people with Parkinson’s disease, who were evaluated off their medication at preoperative baseline and 6 months after deep brain stimulation implantation. Brain activity related to verbal and visuospatial working memory tasks was recorded using electroencephalography at both baseline and during follow-up evaluations conducted under stimulation. The follow-up assessments were carried out following 3-week periods of either omnidirectional or directional stimulation, applied in a randomized, double-blind, crossover design. Average postoperative working memory performance remained stable at the group level regardless of the stimulation condition for both verbal and visuospatial working memory tasks. However, at the individual level, higher alpha and beta power at baseline was associated with slower visuospatial working memory reaction time at follow-up. Additionally, reductions in theta and beta power during stimulation at follow-up correlated with better verbal working memory accuracy during the task and compared with baseline, respectively. These findings suggest that task-related electroencephalography may provide candidate physiological markers of individual working memory trajectories after subthalamic deep brain stimulation. Oscillatory brain activity may help to characterize stimulation-related cognitive variability beyond motor outcomes, but these exploratory findings require validation in larger cohorts before they can inform stimulation programming or closed-loop treatment strategies. Registration: ClinicalTrials.gov: NCT03548506

## Teaser

Keute *et al*. report that verbal and visuospatial working memory outcomes after deep brain stimulation in Parkinson’s disease were linked to baseline cortical activity and stimulation-related spectral changes, suggesting candidate physiological markers of individual cognitive trajectories after treatment.

## Graphical Abstract

**Graphical Abstract:**
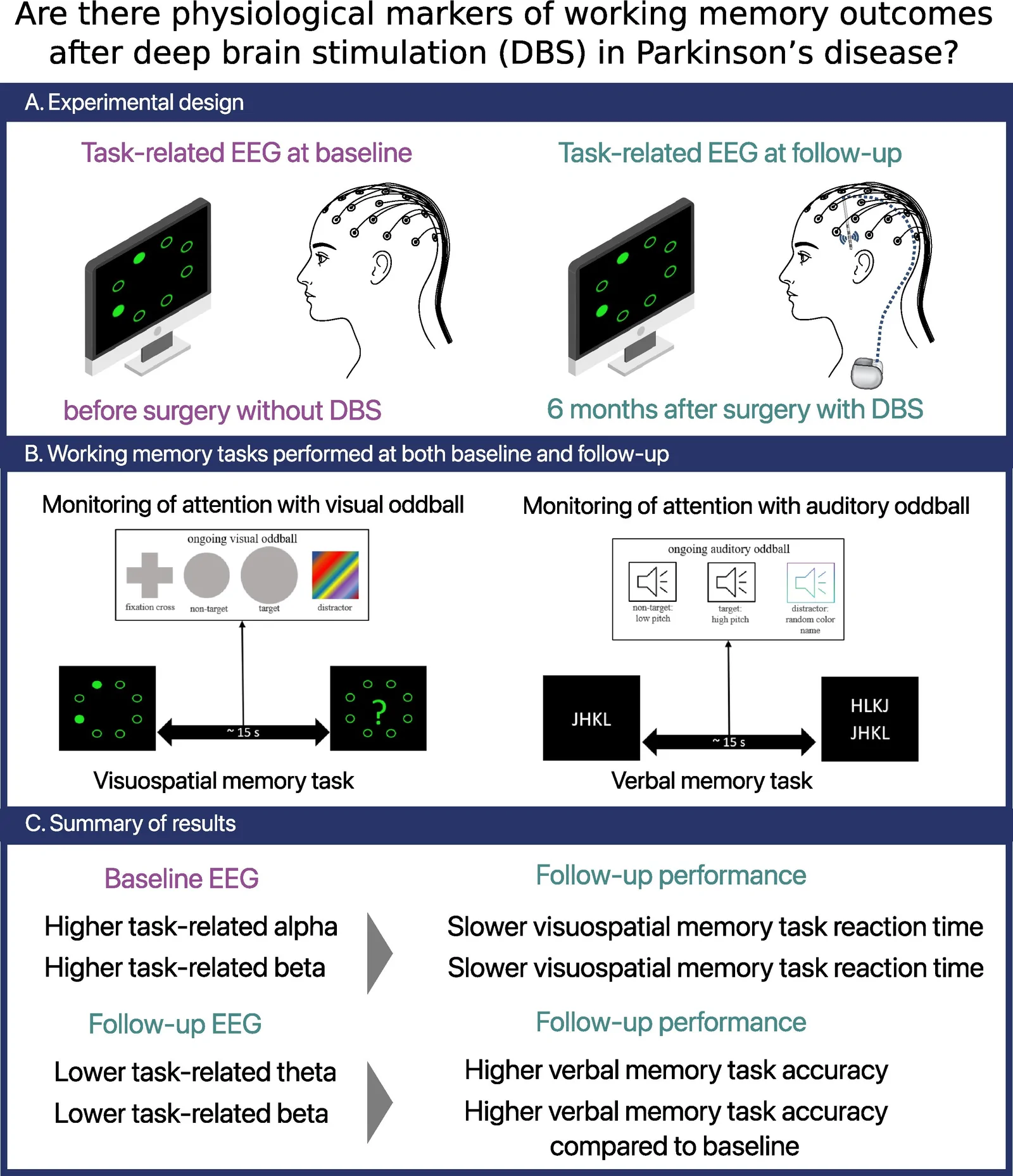

## Introduction

Neural oscillations have become essential for revealing the mechanisms behind both normal behaviour and pathological states in people with Parkinson’s disease. In particular, beta oscillations have been identified as a significant factor in Parkinson’s disease motor impairment,^1^ and their therapeutic modulation by medication and deep brain stimulation (DBS) has been studied for both subcortical^2^ and cortical areas.^3^ Specifically, previous work in people with Parkinson’s disease revealed that subthalamic nucleus (STN)-DBS significantly modifies network dynamics by enhancing movement-related desynchronization in the upper alpha and beta frequency bands across bilateral parietal, sensorimotor, premotor, supplementary motor and prefrontal areas. This extended desynchronization was associated with improved clinical motor outcomes and faster reaction times (RTs).^3^ Given these insights and the integrative role of the STN in motor and cognitive networks,^4^ it is warranted to explore the effects of this network modulation on cognitive functions, such as working memory in Parkinson’s disease. To this end, identifying brain–behaviour relationships may pave the way for STN-DBS beyond its established role in alleviating motor symptoms by engaging distributed brain networks^5^ and targeting specific components involved in cognitive functions.^6^

Recent work explored the mechanisms behind cognitive impairment in Parkinson’s disease by intraoperative recordings in cortical and subcortical areas and demonstrated oscillatory beta power modulations during a working memory *n*-back task.^7^ Specifically, participants with mild cognitive impairment exhibited a smaller decrease in task-related beta power compared with those with less severe cognitive impairment in specific phases of the task, despite no significant differences in task performance. In this context, STN-DBS may modulate working memory-related cortical beta desynchronization, similar to its well-documented effects on movement-related beta desynchronization.^3^ However, the direction and magnitude of cognitive effects after STN-DBS are likely to depend on baseline cognitive status, stimulation location, task demands and the cortical networks engaged by stimulation. Therefore, stimulation-related oscillatory changes should be examined as candidate physiological markers rather than as direct evidence of cognitive improvement.

In evaluating the effects of DBS on cognition, it is essential to consider the pathophysiological baseline state^8^ and the timing of evaluation. Immediate postsurgery assessments may be influenced by the surgery and insertional effect of implantation,^9^ while delayed evaluations risk confounding factors like ageing and disease progression,^10^ thus suggesting an intermediate evaluation period.

Furthermore, the application of directional steering (dDBS) to the induced electrical field with segmented electrodes may also be of relevance. Recent research indicates that the fine-tuning of current intensity during parameter titration for rigidity resolution^11^ and the subsequent application of dDBS can mitigate a potential decline in executive function associated with circular/omnidirectional stimulation (oDBS) using classical ring electrodes.^12^ These observations support the need to compare stimulation modes after clinically relevant stimulation periods, while recognizing that experimental working memory tasks cannot capture the full range of cognitive domains affected by subthalamic stimulation.

In this study, we built on this previous research by combining STN-DBS with surface EEG during spatial and verbal working memory tasks, comparing dDBS and oDBS electrical field steering 6 months after implantation. This approach aimed to better understand the impact of this intervention on cognitive task-related oscillatory network characteristics.^13^ Our goal was to identify candidate physiological biomarkers of individual working memory outcomes after stimulation, with potential relevance for future therapeutic strategies, such as closed-loop approaches. Based on findings in the motor^3^ and cognitive domains,^7^ we hypothesized that a greater reduction in task-related beta power during DBS would be associated with better working memory performance.

## Materials and methods

**Figure 1:**
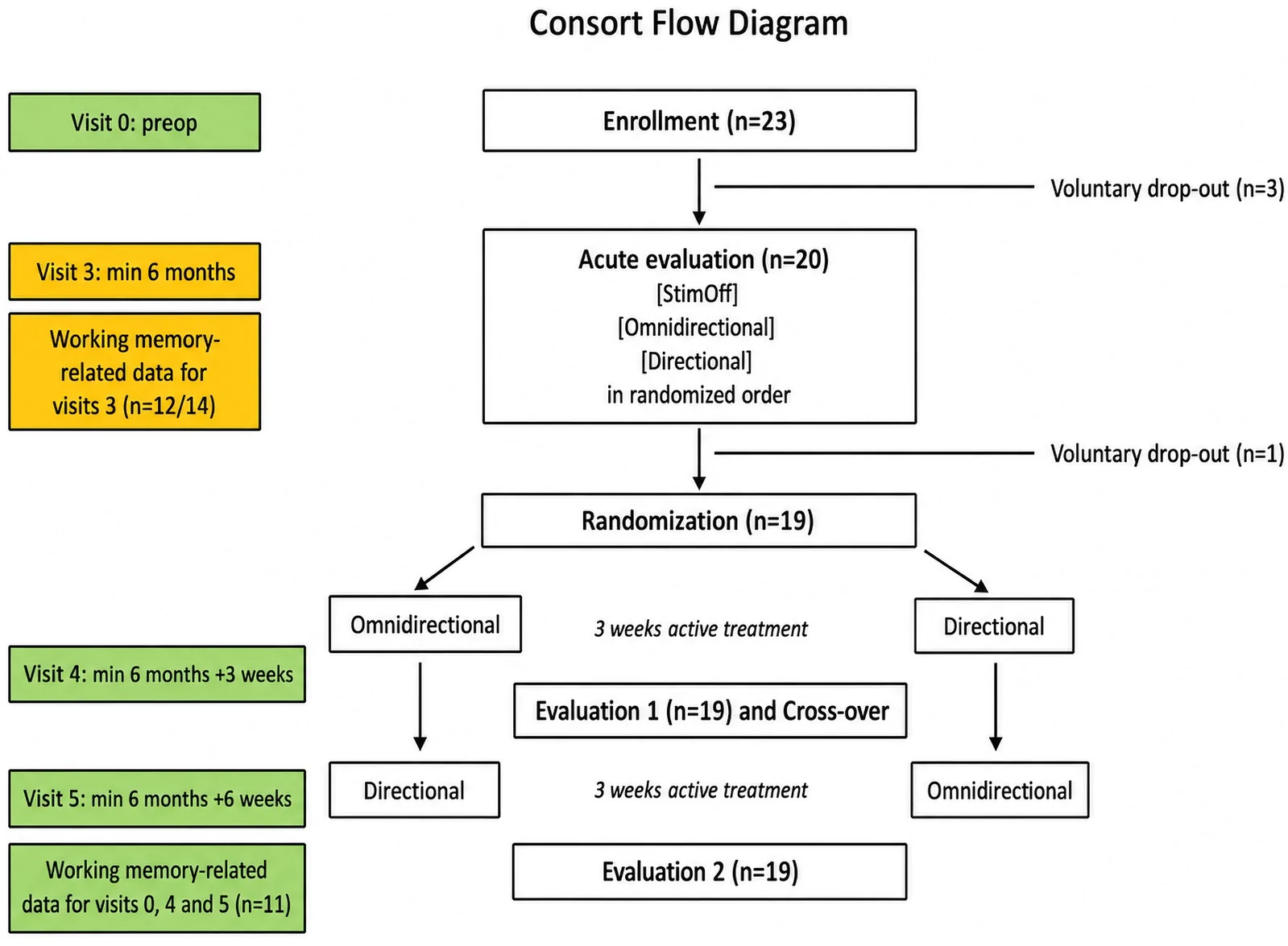
**Consort flowchart.** Consort flowchart of participant enrolment, randomization and assessments of the present study (see also Gharabaghi *et al*.^14^ for the primary study). This prospective, randomized clinical trial included postoperative assessments at a minimum of 6 months after surgery at two stages: acute (within 1 day) and chronic (after 3-week stimulation blocks). The trial followed a double-blind crossover design with medication off. During the acute assessments, performance was compared across the no-stimulation condition (StimOFF), omnidirectional stimulation (oDBS) and directional stimulation (dDBS). During chronic assessments, oDBS and dDBS conditions were compared with each other and with the preoperative baseline. EEG recordings were acquired at all visits indicated in the timeline.

This study is an ancillary analysis of electroencephalographic data acquired during working memory tasks in the context of a prospective, randomized, double-blind, crossover, single-centre clinical trial, i.e. the Subthalamic steering for therapy optimization in Parkinson’s Disease (SANTOP) study (preregistered at ClinicalTrials.gov: NCT03548506). The comprehensive study design is depicted in Fig. 1. The parent trial design, patient recruitment, surgical procedure, randomization, electrode selection, stimulation programming and clinical outcome assessments have been reported previously.^12^ The present manuscript focuses on the working memory tasks and task-related electroencephalographic analyses acquired within this predefined clinical trial framework.

### Patients

As reported in the main study,^12^ all patients who participated in this study were nominated for DBS surgery upon multidisciplinary review based on standard inclusion and exclusion criteria for STN-DBS in Parkinson’s disease,^15^ independent of study participation. Ages eligible for the study were 18–80 years. Exclusion criteria were pregnancy, cognitive impairment (Mini-Mental State Exam < 20), suicidality, psychosis or other severe pathological chronic conditions that might confound treatment effects or interpretation of the data. The study was conducted in accordance with the Declaration of Helsinki and approved by the ethics committee of the Medical Faculty of the University of Tübingen (700/2017B01).^12^

**Figure 2:**
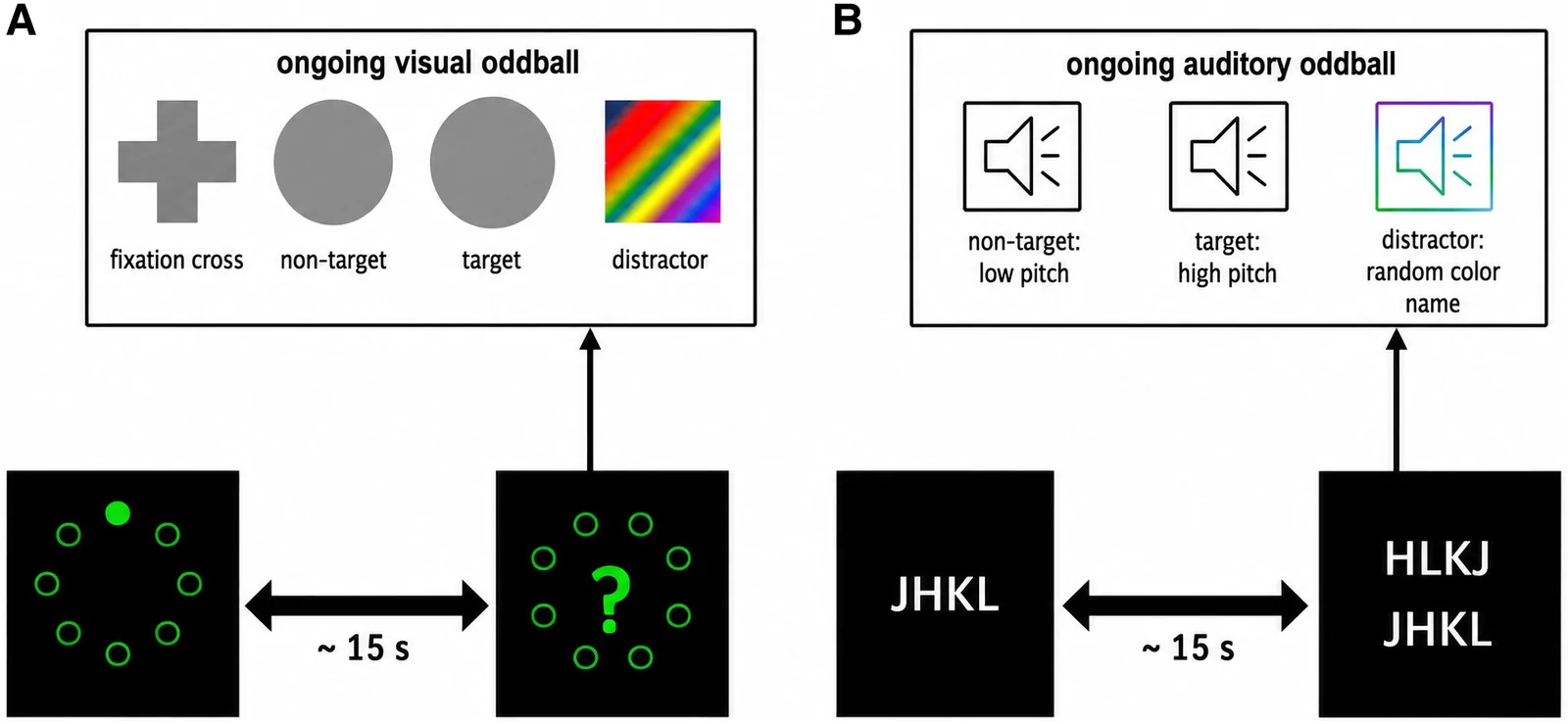
**Experimental procedures.** Illustration of the experimental procedures that assess working memory and attentional processes by integrating (**A**) visual or (**B**) auditory oddball tasks within spatial or verbal delayed response paradigms.

A total of 23 consecutive patients with akinetic-rigid Parkinson’s disease scheduled for STN-DBS were enrolled in the SANTOP study. Written informed consent was obtained from all participants prior to inclusion. Verbal and visuospatial working memory were assessed using the verbal delayed response (VDR)^16^ and spatial delayed response (SDR)^17^ tasks (written in Python, version 2.7.14, Anaconda, Inc.) at baseline before surgery and again at least 6 months after electrode implantation, with concurrent EEG recordings.^18^ Therefore, baseline EEG refers to the task-related recordings acquired before DBS implantation and without any stimulation. EEG was recorded at each of the study visits included in the experimental timeline. Standardized neuropsychological working memory tests were not included in the protocol, and no direct comparison between clinical working memory scores and the experimental tasks was possible. Attentional levels were monitored using auditory and visual oddball tasks embedded within the VDR and SDR tasks, respectively (Fig. 2). In the SDR, a visual oddball stream was presented during the delay period, consisting of frequent non-target stimuli and infrequent target stimuli requiring a button press.^19^ In the VDR, auditory tones were presented in an analogous structure, with rare target tones interspersed among frequent standard tones.^20^ The timing and probability structure followed the fixed protocol and was identical for all participants. These oddball elements served to monitor sustained attention without interfering with the primary working memory manipulation.

Postoperative assessments were conducted 6 months after surgery in both an acute phase (within 1 day) and a chronic phase (following two consecutive 3-week stimulation blocks), in a prospective, randomized, double-blind crossover design under medication-off conditions (Med OFF). During the acute assessments, performance was compared across the no-stimulation condition (StimOFF), oDBS and directional stimulation (dDBS). During the chronic assessments, oDBS and dDBS were compared with each other and with the preoperative baseline. For the directional stimulation condition, stimulation intensity was adjusted to match the total electrical energy delivered by oDBS (Table 1).

**Table 1:** Patient demographics and stimulation parameters

ID	Sex/gender	Age (years)	Disease duration (years)	LEED (mg/day)	Stimulation parameters omnidirectional	Stimulation parameters directional
1	M	65	10	1125	L 3− G+ 2.1 mA 60 µs 130 Hz R 11− G+ 3.7 mA 60 µs 130 Hz	L 3C− G+ 1.4 mA 60 µs 130 Hz R 11B− G+ 2.3 mA 60 µs 130 Hz
2*	F	61	15	869	L 3− G+ 3.3 mA 60 µs 130 Hz R 11− G+ 3.5 mA 60 µs 130 Hz	L 3A− G+ 2.5 mA 60 µs 130 Hz R 11C− G+ 2.5 mA 60 µs 130 Hz
3	M	58	11	1250	L 3− G+ 1.3 mA 60 µs 130 Hz R 11− G+2.3 mA 60 µs 130 Hz	L 3C− G+ 0.5 mA 60 µs 130 Hz R 11A− G+ 1.2 mA 60 µs 130 Hz
4*	M	71	15	450	L 3− G+ 2.70 mA 60 µs 130 Hz R 11− G+ 2.20 mA 60 µs 130 Hz	L 3A− G+ 2.2 mA 60 µs 130 Hz R 11A− G+ 2.0 mA 60 µs 130 Hz
5	M	58	9	1205	L 3− G+ 1.5 mA 60 µs 130 Hz R 11− G+ 1.5 mA 60 µs 130 Hz	L 3A− G+ 0.95 mA 60 µs 130 Hz R 11C− G+ 1.05 mA 60 µs 130Hz
6	M	56	6	1293	L 3− G+ 0.8 mA 60 µs 130 Hz R 11− G+ 4.5 mA 60 µs 130 Hz	L 3A− G+ 0.55 mA 60 µs 130 Hz R 11A− G+ 3.1 mA 60 µs 130 Hz
7	M	46	11	1265	L 3− G+ 1.6 mA 60 µs 130 Hz R 11− G+ 3.7 mA 60 µs 130 Hz	L 3A− G+ 1.25 mA 60 µs 130 Hz R 11B− G+ 2.85 mA 60 µs 130 Hz
8*	F	68	12	815	L 3− G+ 2.2 mA 60 µs 130 Hz R 11− G+ 4.0 mA 60 µs 130 Hz	L 3A− G+ 1.8 mA 60 µs 130 Hz R 11C− G+ 2.7 mA 60 µs 130 Hz
9	F	53	14	375	L 3− G+ 2.8 mA 60 µs 130 Hz R 11− G+ 2.9 mA 60 µs 130 Hz	L 3A− G+ 2.05 mA 60 µs 130 Hz R 11B− G+ 1.95 mA 60 µs 130 Hz
10	F	75	13	725	L 3− G+ 4.7 mA 60 µs 130 Hz R 11− G+ 4.7 mA 60 µs 130 Hz	L 3B− G+ 3.35 mA 60 µs 130 Hz R 11B− G+ 3.35 mA 60 µs 130 Hz
11	F	59	18	525	L 3− G+ 1.1 mA 60 µs 130 Hz R 11− G+ 1.5 mA 60 µs 130 Hz	L 3B− G+ 0.75 mA 60 µs 130 Hz R 11C− G+ 1.00 mA 60 µs 130 Hz
12	M	61	6	441	L 3− G+ 2.7 mA 60 µs 130 Hz R 11− G+ 2.8 mA 60 µs 130 Hz	L 3C− G+ 2.6 mA 60 µs 130 Hz R 11A− G+ 1.95 mA 60 µs 130 Hz
13	M	73	10	1033	L 3− G+ 2.5 mA 60 µs 130 Hz R 11− G+ 2.7 mA 60 µs 130 Hz	L 3B− G+ 1.7 mA 60 µs 130 Hz R 11B− G+ 1.85 mA 60 µs 130 Hz
14	M	71	8	875	L 3− G+ 3.0 mA 60 µs 130 Hz R 11− G+ 2.8 mA 60 µs 130 Hz	L 3C− G+ 2.1 mA 60 µs 130 Hz R 11A− G+ 1.8 mA 60 µs 130 Hz
15	M	67	12	815	L 2− G+ 1.7 mA 60 µs 130 Hz R 10− G+ 2.2 mA 60 µs 130 Hz	L 2C− G+ 1.1 mA 60 µs 130 Hz R 10B− G+ 1.4 mA 60 µs 130 Hz
16	M	67	18	960	L 3− G+ 2.8 mA 60 µs 130 Hz R 11− G+ 3.3 mA 60 µs 130 Hz	L 3B− G+ 2.1 mA 60 µs 130 Hz R 11B− G+ 2.3 mA 60 µs 130 Hz
17	F	52	8	708	L 3− G+ 2.3 mA 60 µs 130 Hz R 11− G+ 2.1 mA 60 µs 130 Hz	L 3C− G+ 1.65 mA 60 µs 130 Hz R 11C− G+ 1.5 mA 60 µs 130 Hz
18	M	50	9	859	L 3− G+ 2.7 mA 60 µs 130 Hz R 11− G+ 0.9 mA 60 µs 130 Hz	L 3A− G+ 1.9 mA 60 µs 130 Hz R 11B− G+ 0.5 mA 60 µs 130 Hz
19	M	67	13	1050	L 3− G+ 2.7 mA 60 µs 130 Hz R 11− G+ 2.5 mA 60 µs 130 Hz	L 3C− G+ 1.8 mA 60 µs 130 Hz R 11C− G+ 1.7 mA 60 µs 130 Hz

The table shows the patient demographics and stimulation parameters (modified from Gharabaghi *et al*.^14^). LEED indicates the levodopa equivalent dose during the crossover phase. The asterisk ‘*’ indicates patients in whom a reprogramming was necessary during the crossover phase: ID2: the calculated directional stimulation was L 3A− G+ 2.1 mA 60 µs 130 Hz, R 11C− G+ 2.2 mA 60 µs 130 Hz; patient reported bradykinesia and had rigidity and freezing of gait with directional stimulation during the crossover phase so that we needed to increase the stimulation intensity.

ID4: the calculated directional stimulation was L 3A− G+ 1.85 mA 60 µs 130 Hz, R 11A− G+ 1.55 mA 60 µs 130 Hz; patient reported bradykinesia and had rigidity with directional stimulation during the crossover phase so that we needed to increase the stimulation intensity.

ID8: the calculated directional stimulation was L 3A− G+ 1.6 mA 60 µs 130 Hz, R 11C− G+ 2.6 mA 60 µs 130 Hz; patient reported worsening of motor fluctuations so that we needed to increase the stimulation intensity.

Four patients discontinued participation for personal reasons unrelated to the study. Nineteen patients completed the full crossover treatment evaluation periods. After applying quality control procedures, complete datasets were available for 14 patients for acute VDR, 12 patients for acute SDR and 11 patients for chronic VDR and SDR. Participant flow throughout the study is summarized in Fig. 1, and patient demographics are presented in Table 1.

### Surgical procedure

The surgical procedure was performed as previously published for the parent SANTOP trial.^12^ In brief, subthalamic nucleus targeting was based on preoperative magnetic resonance imaging^21^ and intraoperative neurophysiological confirmation.^22^ Electrophysiological recordings were used to confirm the target region, and the final lead position was selected to place segmented contacts within the subthalamic region showing prominent beta-band activity.^23^ No surgical complications occurred in the present cohort.

### Study design

The randomized, double-blind crossover study design was performed as previously published for the parent SANTOP trial.^12^ Briefly, patients were enrolled preoperatively and underwent postoperative visits for stimulation titration and treatment evaluation. All assessments were performed after overnight withdrawal of dopaminergic medication. Patients and evaluators were blinded to stimulation condition throughout the study.

At the 6-month visit, stimulation-off, omnidirectional and directional stimulation were assessed in randomized order after short-term reprogramming. Thereafter, patients underwent two consecutive 3-week treatment blocks with omnidirectional or energy-equivalent directional stimulation in randomized crossover order. Working memory performance and task-related EEG were assessed during these visits. The study epochs are summarized in Fig. 1.

### Identification of optimal contact and stimulation parameters

Stimulation titration was performed approximately 2 months after surgery during two blinded postoperative visits (V1 and V2) following overnight medication withdrawal.^11^ The procedures included monopolar testing of omnidirectional and segmented contacts to identify the stimulation level and direction with the best clinical effect on rigidity, using standard stimulation parameters of 130 Hz and 60 μs at matched total electrical energy of oDBS and dDBS.^24^ The detailed protocol for determining optimal contacts, stimulation direction and therapeutic windows is reported in full elsewhere.^12^ For the present study, only the resulting stimulation programmes were used for the acute (STIM OFF, directional and omnidirectional) and chronic (directional versus omnidirectional) assessments (Table 1).

### Treatment evaluation and outcome measures

Working memory tasks (VDR^16^ and SDR^17^) with embedded visual^19^ and auditory oddball components,^20^ together with task-related EEG recordings,^18^ were obtained during the acute V3 visit (STIM OFF, directional and omnidirectional) and the chronic V4/5 crossover assessment (directional versus omnidirectional). Randomization and allocation procedures followed the parent study. Additional motor and non-motor assessments performed at these visits are reported separately^12^ and are not part of the present analysis. Accordingly, no inference on comprehensive cognitive outcome or motor treatment response is made from the present task-based EEG analysis.

### Visuospatial working memory

The visuospatial working memory task was based on an adapted version of the SDR paradigm (Fig. 2A).^17^ At the start of each trial, two of eight spatial positions arranged in a circular layout were highlighted for 150 ms and had to be memorized. After a fixed 15 s delay interval, a response display appeared for 5 s, during which participants indicated the two remembered positions using an eight-button response pad. Each run contained 20 trials and lasted approximately 7 min.

A continuous visual oddball task^19^ was presented during the delay interval. Three stimulus categories were shown: a small white circle (target), a large white circle (non-target) and a colourful rectangle (distractor). Each stimulus was preceded by a fixation cross that served as a size reference. Participants were instructed to press a response button whenever a target stimulus appeared. Target stimuli were presented infrequently relative to non-targets, following the fixed oddball structure implemented in the task software, which was identical for all participants. Oddball events were interleaved with the SDR delay periods but did not overlap with the encoding or response cues.

### Verbal working memory

The verbal working memory task was adapted from the delayed response paradigm described (Fig. 2B).^16^ Each trial began with the presentation of four letters in a fixed sequence that participants were instructed to memorize. After a 15 s delay interval, two four-letter sequences were shown. One sequence matched the memorized order, and the other contained the same letters rearranged. Participants selected the correct sequence within a 5 s response window. The task consisted of 20 trials with a total duration of approximately 7 min.

An auditory oddball task^20^ was presented continuously during the delay interval. Standard tones served as non-target stimuli, higher-pitched tones served as target stimuli requiring a button press, and spoken colour words functioned as distractor stimuli. Target stimuli were presented infrequently relative to non-targets, following the fixed oddball structure implemented in the task software, which was identical for all participants. Oddball events were interleaved with the VDR delay interval but did not overlap with encoding or response phases.

### EEG data acquisition

In both experiments, EEG was recorded with a BrainAmp amplifier system (Brain Products GmbH, Germany) at a nominal acquisition sampling rate of 5000 Hz, from 64 Ag/AgCl contacts arranged according to the international 10-5 system (Easycap GmbH, Herrsching, Germany; online reference channel: FCz grounded to AFz). Continuous data were subsequently downsampled during preprocessing as described below.

### Data analysis

The data were analysed using custom scripts based on the MNE toolbox (version 0.23.0) in Python (version 3.8.5, Anaconda, Inc.). Statistical analyses were performed using R version 3.6.3, and the statistical packages lme4 and lmerTest. Data visualizations were generated in Python using the matplotlib and ggplot2 packages. The analysis was exploratory and was not powered a priori for stimulation-mode effects on electroencephalographic working memory outcomes.

### Behavioural data analysis

To examine the cognitive performance of the patients during the working memory tasks, we calculated the response accuracy and response time (RT) preoperatively, as well as in the oDBS and dDBS conditions.

In the VDR task, accuracy meant pressing the correct button that matched the previously shown sequence of letters within 3 s of the response display appearing. If this criterion was met for a trial, the RT, defined as the time difference between the response cue and the button press, was also computed.

In contrast to the VDR task, which required subjects to press only one button, the SDR task required two button presses. Consequently, it was possible in each SDR trial to obtain zero, one or two positions correct. Accuracy in each trial was thus awarded either 0, 0.5 or 1 point. For both tasks, the obtained points were divided by the total number of trials, resulting in the response accuracy ratio. In the same manner, the mean RT was computed by averaging across the RTs of the valid trials (for the SDR task: first response).

Analogous to the delayed response task evaluations, the oddball tasks were evaluated based on accuracy and RT. In this case, a trial represents the display of the target oddball. If the subject reacted correctly by pressing the right button on the console, a correct response and its RT were recorded.

### EEG data analysis

EEG preprocessing followed established procedures used in previous work from our group^18^ and combined the preprocessing framework implemented for the present study with additional artefact control steps. Continuous data were downsampled to 250 Hz before spectral analysis. A notch filter was applied to suppress powerline interference, followed by a 1–40 Hz bandpass filter to remove slow drifts and high-frequency noise, including stimulation-related artefacts. All filters were implemented as zero-phase finite impulse response filters.

All recordings were visually inspected to identify excessive noise or technical artefacts. Channels with persistent noise were removed and later interpolated using spherical splines.^25^ Surface Laplacian computation was performed to enhance spatial specificity and reduce the influence of volume conduction.^26,27^ Epochs containing amplifier saturation, abrupt voltage steps or muscle artefacts were rejected prior to further analysis.

Ocular and blink-related artefacts were identified and removed using independent component analysis. Components reflecting ocular activity, defined by their characteristic scalp topography and time course, were excluded before reconstructing the cleaned EEG signal. Remaining epochs were visually inspected again to confirm the absence of residual artefacts.

For spectral analysis, data were segmented into epochs time-locked to the target cue of each trial. Three-second windows before and after the cue were extracted. Time–frequency decomposition was performed using the spectrogram function from the scipy.signal package.^28^ Spectrogram coefficients were log-transformed and multiplied by 10 to obtain decibel values. Baseline correction was performed using the 2 s pre-cue interval for each task. Median power values were computed for delta (1–4 Hz), theta (4–8 Hz), alpha (8–12 Hz) and beta (13–30 Hz) bands across frequencies, channels and time, yielding one normalized estimate per band. In addition, median spectral responses per channel (across time) and per time window (across channels) were obtained. These band-limited features served as input to the subsequent statistical analyses.

### Statistical analysis

In this study, we report analyses of VDR and SDR performance and related EEG signatures across baseline, acute and chronic assessments. For behavioural analyses, task RT and task accuracy were normalized against the preoperative baseline value by subtraction where appropriate. Specifically, task-related spectral power values were normalized within subjects by subtracting each participant’s own power estimate from the mean power during the prestimulus interval of the corresponding task for each frequency band and electrode. This subtraction isolates task-related changes from interindividual differences in absolute power and yields subject-specific modulation values that can be directly compared across stimulation conditions and visits. The normalized values were used as input to the mixed-effects models.

To assess the effect of the stimulation condition on each of the behavioural and physiological outcomes, linear mixed models were applied with stimulation condition (dDBS and oDBS) as a within-subject factor and included random intercepts for each patient to account for repeated measurements. Model coefficients were tested against zero by *t*-tests with Satterthwaite correction for degrees of freedom. Furthermore, the models were contrasted with a null model comprising solely the intercept term, without any stimulation effect, and Bayes factors were calculated in accordance with the Bayes–Schwarz Information Criterion. To ascertain whether behavioural outcomes were associated with physiological patterns, Pearson’s correlation coefficients were calculated between task-related spectral responses at baseline without stimulation and the change-to-baseline in task parameters (accuracy/RT). Since there were no significant behavioural differences between conditions, we calculated correlations across both dDBS and oDBS sessions. This entailed pairing each patient’s baseline values with both follow-up stimulation conditions, which did not increase the number of independent participants and was therefore interpreted as an exploratory condition-level analysis.

## Results

### Stable working memory performance during acute and chronic assessments

During the acute assessments (V3), there were no differences in accuracy and RT between StimOFF, oDBS and dDBS for the VDR (all *P* > 0.13, *χ*^2^ < 3.9) and SDR (all *P* > 0.2, *χ*^2^ < 3.3) tasks. However, there was a time effect independent of the stimulation condition, i.e. the task performance improved from the first to the third session for the acute VDR (accuracy: *P* = 0.002, *χ*^2^ = 12.146; RT: *P* = 0.008, *χ*^2^ = 9.715) and SDR (RT: *P* = 0.041, *χ*^2^ = 6.403) tasks, which was not present during the chronic assessments (all *P* > 0.08, *χ*^2^ < 3.08). Similarly, there were no performance differences between StimOFF, oDBS and dDBS for the auditory (all *P* > 0.06, *χ*^2^ < 7.52) and visual (all *P* > 0.28, *χ*^2^ < 2.53) oddball tasks, apart from a time effect on the accuracy in the acute auditory oddball task (*P* = 0.039, *χ*^2^ = 6.467), which was not present during the chronic assessment (*P* = 0.112, *χ*^2^ = 2.531).

**Figure 3:**
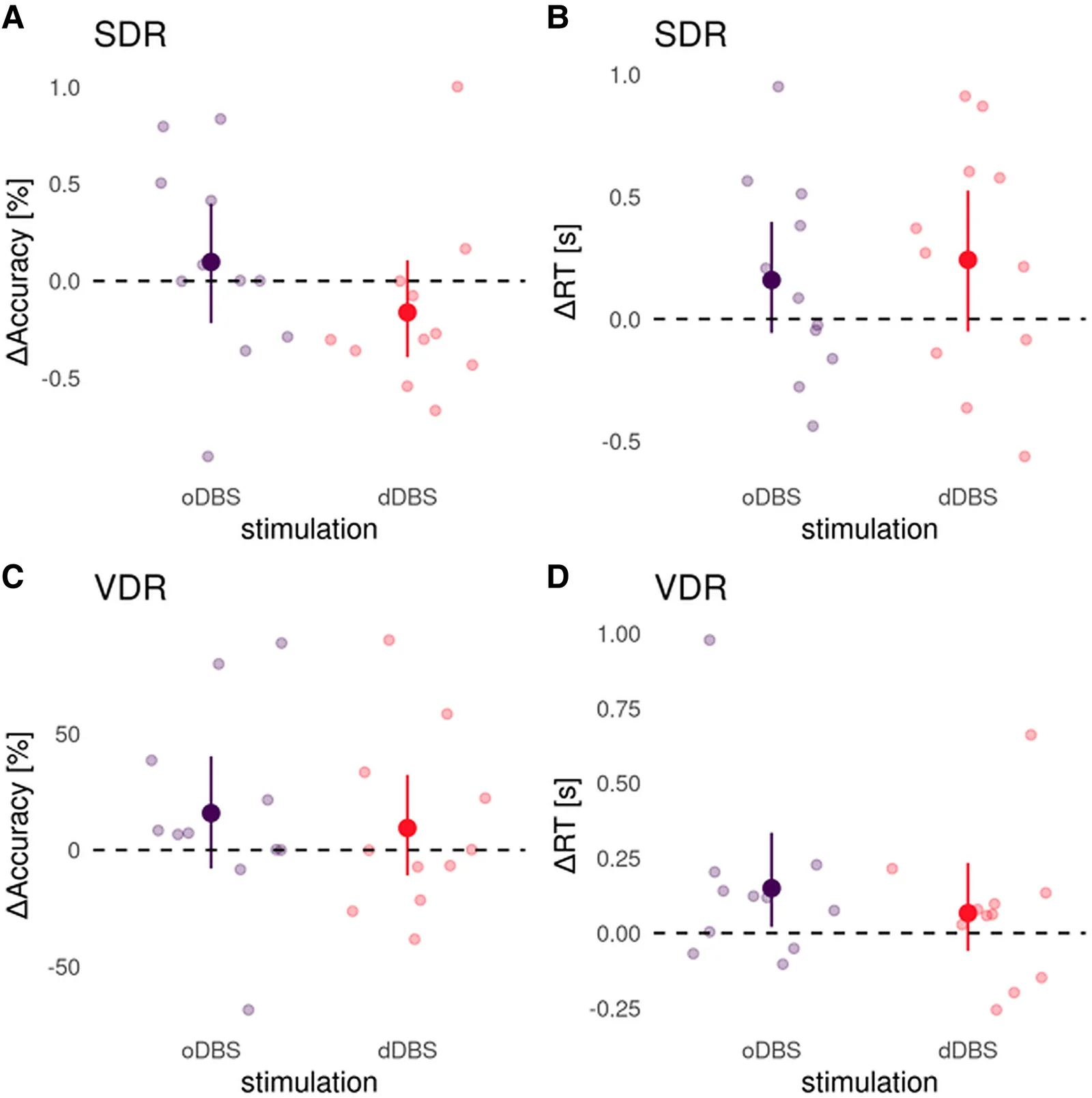
**Task performance.** (**A**) Accuracy and (**B**) response time (RT) from the spatial delayed response (SDR) task, (**C**) accuracy and (**D**) RT from the verbal delayed response (VDR) task in the omnidirectional (oDBS) and directional (dDBS) stimulation conditions (SDR, *n* = 11; VDR, *n* = 11). Values are expressed as the difference from the preoperative baseline; small dots represent individual patients and large dots with whiskers represent the group mean with bootstrapped 95% confidence intervals. Paired *t*-tests were used to compare stimulation conditions and baseline versus follow-up performance. During the chronic assessments (V4/5), there was no significant effect of stimulation condition on accuracy (SDR: *t* = −1.76, *P* = 0.109; VDR: *t* = −0.49, *P* = 0.634) or RT (SDR: *t* = 0.84, *P* = 0.420; VDR: *t* = −1.64, *P* = 0.132). Bayes factors indicated substantial evidence for the null (all BF01 > 1). There were also no differences between baseline and follow-up for accuracy (SDR: *t* = −0.25, *P* = 0.806; VDR: *t* = 1.21, *P* = 0.253) or RT (SDR: *t* = 1.58, *P* = 0.144; VDR: *t* = 1.38, *P* = 0.195).

During the chronic assessments (V4/5), there was no significant effect of stimulation condition on accuracy (SDR: *t* = −1.76, *P* = 0.109, BF01 = 1.06; VDR: *t* = −0.49, *P* = 0.634, BF01 = 4.12) or RT (SDR: *t* = 0.84, *P* = 0.420, BF01 = 3.22; VDR: *t* = −1.64, *P* = 0.132, BF01 = 1.27) (Fig. 3). Moreover, there were no performance changes between the preoperative baseline assessments and the accuracy (SDR: *t* = −0.25, *P* = 0.806; VDR: *t* = 1.21, *P* = 0.253) and RT (SDR: *t* = 1.58, *P* = 0.144; VDR: *t* = 1.38, *P* = 0.195) at the follow-up assessments. The median accuracy was different between visuospatial and verbal tasks, i.e. 25% in the SDR task (range 3–45%) and 75% in the VDR task (range 30–100%) and showed a large variability within each task. The mean RT was 1.73 ± 0.53 s in the SDR task and 2.47 ± 0.53 s in the VDR task.

### Task-related oscillations as candidate markers for working memory performance

**Figure 4:**
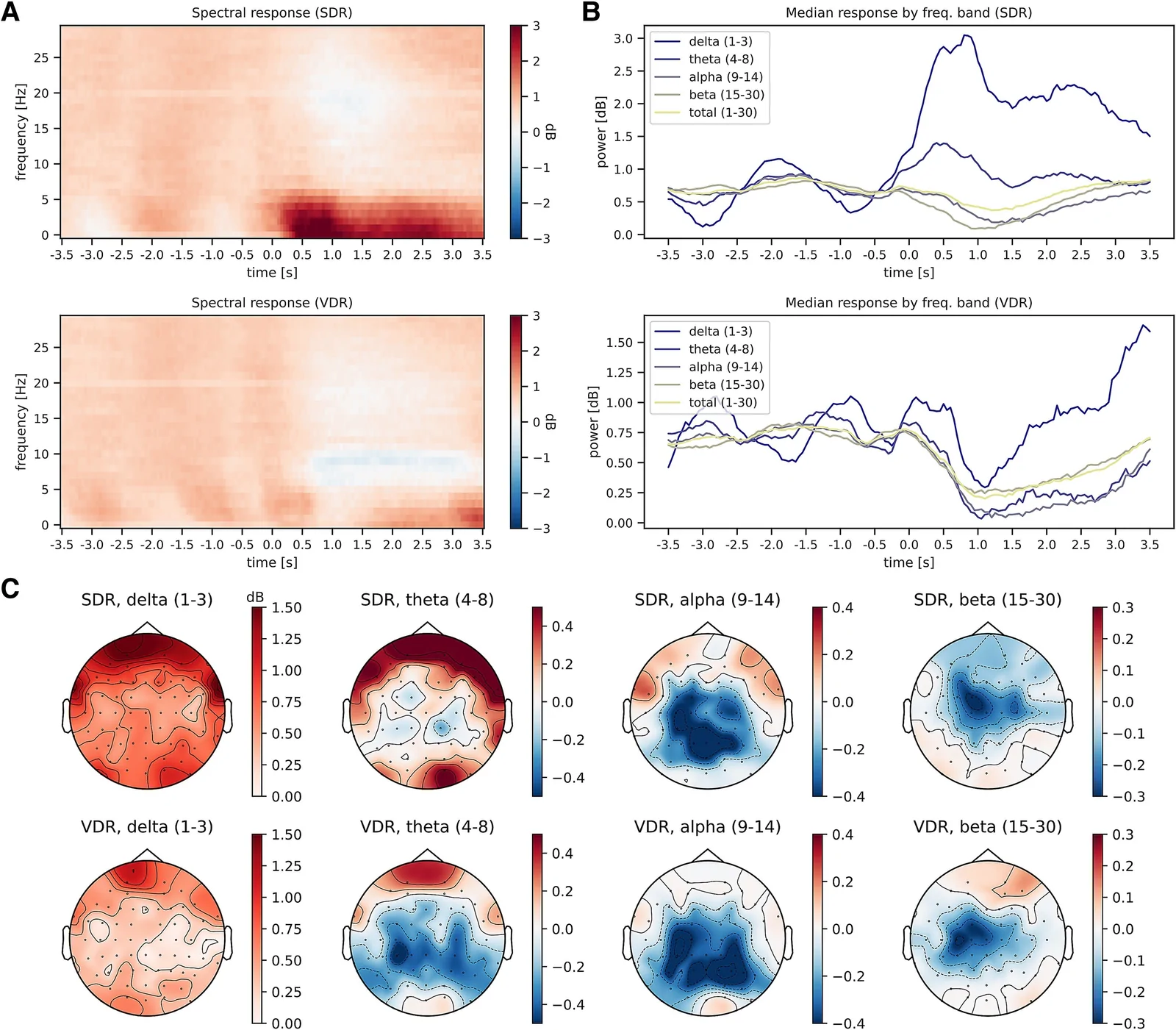
**Spectral responses.** (**A**) Time- and frequency-resolved spectral responses to target cues in the SDR and VDR tasks (SDR, *n* = 11; VDR, *n* = 11, shown as the median across patients, stimulation conditions and EEG electrodes. (**B**) Median spectral power within each frequency band of interest (delta, theta, alpha and beta) and total power. (**C**) Topographies per frequency band (median across the second half of the epoch after cue onset) illustrate a task-related increase in prefrontal delta and theta power and a decrease in frontoparietal theta, alpha and beta power. Across stimulation conditions, only the delta band differed significantly from zero (SDR: 1.58 dB, *t* = 6.1, *P* < 0.001; VDR: 0.46 dB, *t* = 2.3, *P* = 0.043); no other band showed significant modulation (all |*t*| < 2.1, *P* > 0.07). Repeated-measures ANOVAs showed no differences in spectral responses across stimulation conditions (SDR: all *F* < 1.2, all *P* > 0.33, all BF01 > 9.7; VDR: all *F* < 0.7, all *P* > 0.50, all BF01 > 15). One-sample *t*-tests were used to assess spectral responses relative to zero. Heatmap scale bars display spectral power in decibels (dB).

The physiological assessments focused on the chronic assessments, since the acute assessments showed an improved task performance from session to session independently of the stimulation condition, which was not present during the chronic assessments. The spectral response did not differ between stimulation conditions (SDR: all *F* < 1.2, all *P* > 0.33, all BF01 > 9.7; VDR: all *F* < 0.7, all *P* > 0.50, all BF01 > 15). Across conditions, the delta-band response was significantly different from zero (SDR: 1.58 dB, *t* = 6.1, *P* < 0.001; VDR: 0.46 dB, *t* = 2.3, *P* = 0.043), but not in any other frequency band (all |*t*| < 2.1, all *P* > 0.07). As the spectral responses of the stimulation conditions were not different, the respective EEG recordings were pooled for exploratory further analysis. The spectral responses to target cues in both VDR and SDR tasks, averaged across patients and conditions, showed a task-related increase in prefrontal oscillatory activity, particularly in the delta and theta frequency bands, and a decrease in frontoparietal oscillatory activity, particularly in the theta, alpha and beta frequency bands (Fig. 4).

**Figure 5:**
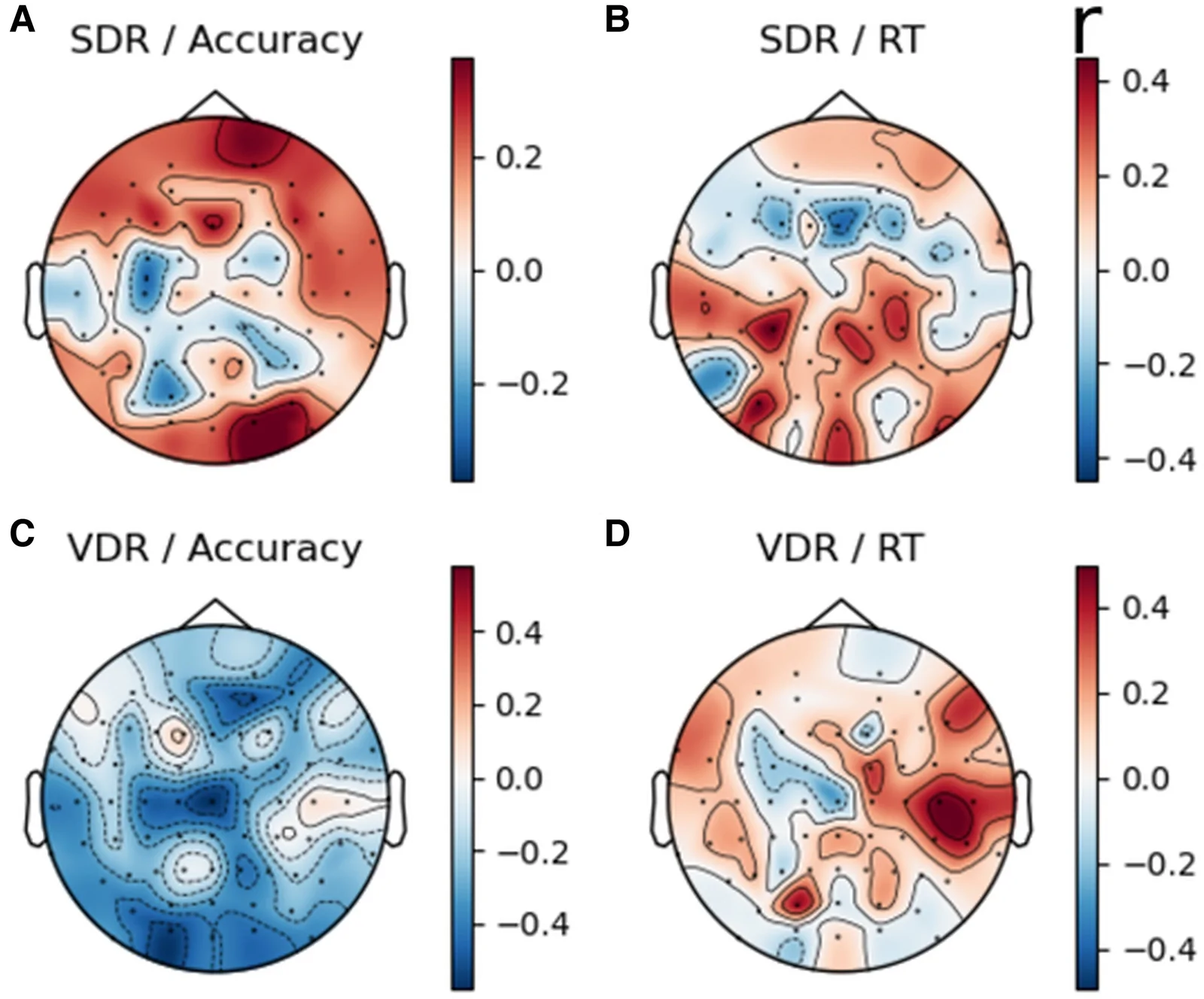
**Spectral distribution of brain–behaviour correlations.** Spatial distribution of beta-band Pearson’s correlations across EEG electrodes at baseline recordings and performance changes from baseline to the 6-month follow-up for (**A**) accuracy and (**B**) reaction time (RT) in the spatial delayed response (SDR, *n* = 11) task and for (**C**) accuracy and (**D**) RT in the verbal delayed response (VDR, *n* = 11) task. Warm colours indicate positive correlations and cool colours indicate negative correlations. A significant positive correlation between baseline beta power and SDR RT was observed (*r* = 0.557, *P* = 0.005), meaning that higher task-related beta activity at baseline (without DBS) predicted slower RT at follow-up (with DBS). For the VDR task, distributed cortical beta desynchronization at baseline corresponded with higher follow-up accuracy (*r* = −0.314, *P* = 0.155), although this association did not reach statistical significance. Each map shows correlation coefficients (*r*) per electrode, with stronger colour saturation reflecting larger absolute values.

The search for candidate oscillatory markers of working memory performance revealed the following findings: The task-related oscillatory power during the presurgical baseline evaluation without stimulation was associated with changes in working memory performance from baseline to the 6-month follow-up evaluation. There was a positive relationship between alpha (*r* = 0.441, *P* = 0.040) and beta (*r* = 0.557, *P* = 0.005) power at baseline and the change in RT in the SDR task. Specifically, the higher the beta power in the sensorimotor cortex, the slower the RT at the follow-up assessment (Fig. 5). In addition, distributed cortical desynchronization in the alpha (*r* = −0.367, *P* = 0.093) and beta (*r* = −0.314, *P* = 0.155) band at baseline corresponded with higher accuracy during the VDR task at the follow-up assessment, although this association did not reach statistical significance.

**Figure 6:**
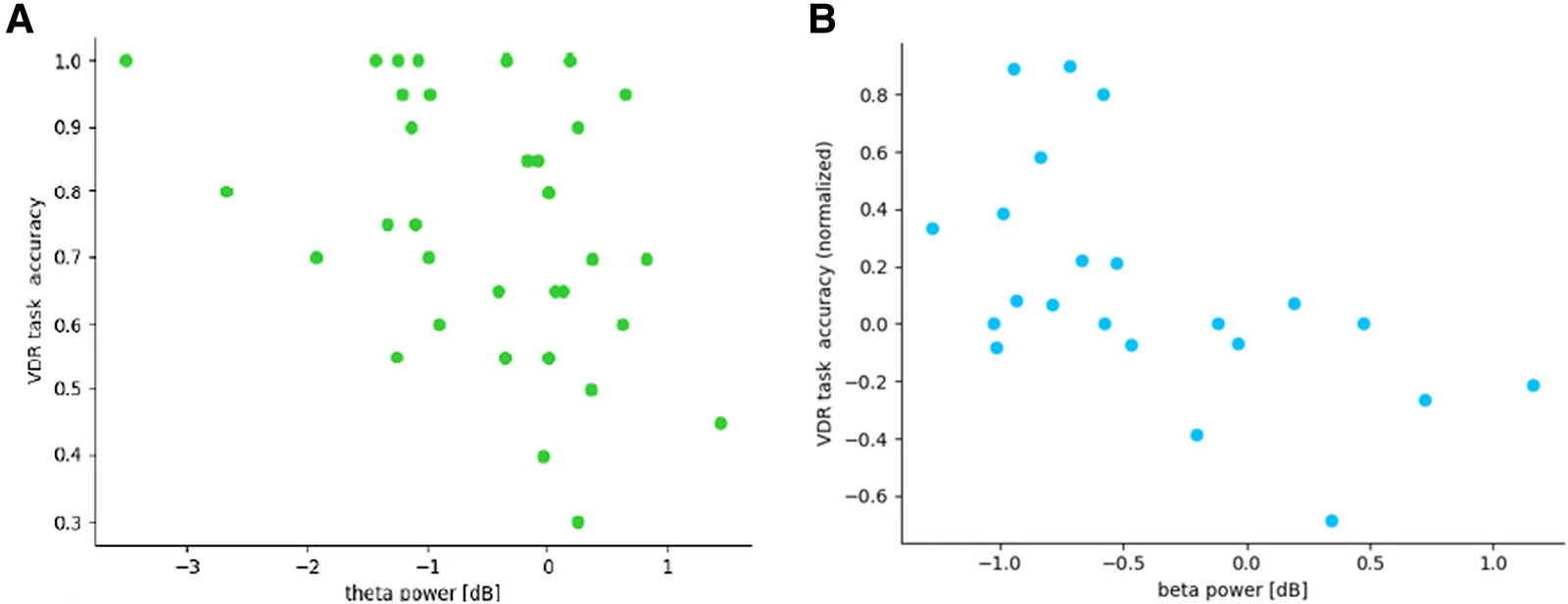
**Brain–behaviour correlations.** (**A**) Task-related desynchronization in the theta band during stimulation 6 months after surgery correlated with higher verbal delayed response (VDR) accuracy during the task. Pearson’s correlation: *r* = −0.406, *P* = 0.019, *n* = 11. (**B**) Task-related desynchronization in the beta band during stimulation correlated with the improvement in VDR accuracy from the preoperative baseline to the 6-month follow-up. Pearson’s correlation: *r* = −0.554, *P* = 0.008, *n* = 11. Each dot represents one patient’s mean spectral change versus behavioural measure. Negative values reflect greater task-related desynchronization, and positive values reflect greater synchronization.

Furthermore, the oscillatory power changes during DBS corresponded with the working memory performance at the follow-up assessment. Specifically, more task-related desynchronization in the theta band correlated with higher VDR accuracy during the task (*r* = −0.406, *P* = 0.019). Moreover, more task-related desynchronization in the beta band correlated with higher VDR accuracy in comparison to the preoperative baseline (*r* = −0.554, *P* = 0.008) (Fig. 6). These brain–behaviour correlations were exploratory and were not corrected for multiple comparisons.

## Discussion

The present study offers insights into the effects of STN-DBS on cortical dynamics associated with working memory in Parkinson’s disease patients. Despite the stable working memory performance observed on average across distinct stimulation conditions and compared with baseline, our findings suggest a nuanced role of cortical oscillations in individual differences in working memory outcomes during DBS.

The pronounced improvement across consecutive acute assessments is consistent with short-term familiarization and practice effects. These dynamics occurred independently of stimulation condition, indicating that they reflected exposure to the task rather than stimulation-related changes. In the chronic phase, performance no longer showed systematic drift, consistent with a stabilized level of task proficiency. Because these acute practice-related changes would add variability to the spectral estimates, EEG analyses were restricted to the chronic assessments, where behavioural performance was stable across visits. This approach avoids confounding short-term learning effects with stimulation-related physiological variability.

While the impact of dopaminergic medication on working memory deficits in Parkinson’s disease patients remains unclear,^29,30^ altered activation of the subthalamic nucleus^31^ and abnormal dynamics in the direct and indirect corticostriatal pathways^32^ appear to contribute significantly to these deficits. In this context, DBS of the STN may modulate working memory networks in either direction, depending on stimulation location, network engagement, baseline cognitive status and task demands.^33^

The patients in this study demonstrated a considerable degree of variability within each working memory (WM) task, with performance on the visuospatial task being notably inferior to that on the verbal task. This may be attributed to the greater cognitive load associated with the former, which required two button presses as opposed to a single button press in the latter, and/or the shorter RTs of the SDR as compared with the VDR task. In addition, spatial WM is particularly vulnerable in Parkinson’s disease.^34,35^ However, while some studies have reported impairments in Parkinson’s disease patients,^36-38^ others have found preserved performance,^39^ and even more pronounced deficits in verbal than spatial tasks.^40^

The SDR and VDR paradigms were chosen because they allow precise control of stimulus timing and working memory load, which differs from the constructs captured by standardized neuropsychological working memory instruments. Such clinical working memory tests were not part of the study protocol, so direct comparisons between experimental performance and clinical neuropsychological scores were not possible. Future studies combining experimental paradigms with standardized assessments in the same individuals would help to determine how these measures relate.

No healthy control group was included in this study, and no external normative data are available for the specific SDR and VDR task variants used here. These paradigms were designed as experimental tasks with precise control over stimulus timing and working memory load, which differs from the structure of standardized neuropsychological tests. As a result, direct comparison of baseline performance with healthy control levels is not possible. Future studies that incorporate matched healthy control groups or validated normative reference datasets would allow clearer interpretation of baseline performance and improve the clinical contextualization of the physiological–behavioural associations observed in this cohort.

The observation that working memory performance remained on average stable regardless of stimulation and paradigm suggests that stimulation settings optimized in the parent trial did not produce detectable group-level changes in the experimental verbal and visuospatial working memory tasks used here. This interpretation is restricted to these task outcomes and does not exclude effects on other cognitive domains, such as verbal fluency, executive function or processing speed. Nevertheless, the considerable variability in performance also highlights the necessity of investigating the specific neural substrates and oscillatory patterns associated with cognitive processes, beyond mere motor measures. Along these lines, the most recent review on this topic concludes that while STN-DBS is generally cognitively safe, it may be associated with declines in verbal fluency, executive function and working memory.^41^ These declines were particularly linked to risk factors such as age and preoperative neuropsychological status. The review emphasizes the need for biomarkers and technological advances to explore the potential for neuromodulation to improve cognitive deficits in Parkinson’s disease.

In this context, the present work provides some new insights: Our results suggested that baseline alpha and beta power were associated with subsequent changes in visuospatial WM RT. Higher baseline beta power, particularly in the sensorimotor cortex, was associated with slower SDR RTs at follow-up, suggesting that elevated synchrony may indicate an impaired neural state less amenable to rapid cognitive processing. This aligns with the well-documented role of elevated beta synchronization in motor impairment such as rigidity and bradykinesia in Parkinson’s disease,^1^ but the present data do not establish whether this association reflects cognitive processing, motor response components of the task, or both. However, it can be postulated that for cognitive tasks that rely heavily on motor responses, the benefits of STN-DBS on movement may result in the improvement of aspects of cognitive performance, such as RT.^4^ Conversely, the predictive value of baseline alpha and beta power may also indicate that these patients experience greater cognitive difficulties. This may be due to the excessive neural synchronization that impedes efficient information processing, which has also been observed in other domains such as response inhibition.^42^

This ambiguity could possibly be addressed, at least in part, by the neurophysiological findings during the experiments with regard to WM accuracy. Specifically, the theta and beta desynchronization during stimulation corresponded to better verbal WM accuracy during the task and compared with baseline, respectively, and may reflect more efficient neural processing and resource allocation. This desynchronization may represent a shift from a pathologically rigid neural state towards a more flexible and adaptive network configuration, which could support cognitive performance.^43,44^ In this context, the observed task-related theta desynchronization in prefrontal cortical areas appears to reflect a general mechanism during verbal WM.^45^ In contrast, the cortically distributed beta-related desynchronization (indicating improved performance compared with baseline) seems to be specific to the pathological condition,^3^ suggesting its potential as a candidate biomarker for adaptive neuromodulation.^46^

Learning-related performance changes and general motor improvement after surgery represent potential confounding factors for the interpretation of the electrophysiological associations reported here. The study protocol did not include simultaneous motor recordings during task performance, and clinical motor ratings were acquired at different time points and at a coarser temporal resolution than the working memory tasks and EEG epochs. A direct statistical adjustment for motor state is therefore not feasible within this dataset. Both learning and motor improvement could influence RTs, accuracy or the magnitude of task-related beta modulation. Future studies would benefit from designs that incorporate concurrent kinematic measures during cognitive tasks, stratification by motor state across stimulation conditions or individualized analyses based on electrode location and structural connectivity to dissociate cognitive from motor contributions more effectively.

However, the findings align with the concept that efficient cognitive processing requires a dynamic balance between neural synchronization and desynchronization.^43^ The observed relationship between task-related desynchronization and working memory performance highlights the importance of neural flexibility in supporting cognitive functions.^44^ The results suggest that interventions aimed at enhancing neural desynchronization may influence cognitive outcomes in Parkinson’s disease patients.

The absence of group-level differences between directional and oDBS does not imply a lack of meaningful variability across individuals. Specifically, interindividual variation in electrode placement, structural connectivity and stimulation spread can produce heterogeneous behavioural and physiological responses despite null effects at the group level. The EEG–behaviour associations identified here therefore reflect stable individual differences in task-related spectral modulation rather than stimulation mode-specific effects. These patterns may help to identify candidate physiological markers that track individual cognitive trajectories and could inform future approaches to optimize factors such as electrode targeting or stimulation programming.

The absence of relevant differences in WM outcomes between oDBS and dDBS conditions suggests furthermore that the current parameter settings did not produce detectable differential effects on the experimental working memory outcomes assessed here.^6^ The identification of oscillatory candidate markers offers a potential avenue for future personalized DBS programming studies, where stimulation parameters could be evaluated based on individual neural profiles. This approach may inform the development of closed-loop DBS systems that dynamically adjust stimulation based on real-time cortical activity.^46^

While this study provides valuable insights, further limitations must be acknowledged. The relatively small sample size and the inherent variability in individual patient responses may limit the generalizability of the findings. The electrophysiological analyses were exploratory, and the sample size was determined by the structure of the parent clinical trial, which did not permit an a priori power analysis for the physiological outcomes. No formal correction for multiple comparisons was applied to the spectral–behavioural correlation analyses. These factors constrain the statistical strength of the inferences, and the findings should be interpreted as preliminary markers rather than definitive effects. Furthermore, the current focus on 3-week evaluation periods 6 months after surgery may not fully capture the longer-term adaptations in cortical dynamics and cognitive function. Future studies with larger cohorts and extended follow-up periods are necessary to validate these findings and explore the longitudinal effects of STN-DBS on working memory. Future research should also consider the role of patient-specific factors such as the extent of pre-existing impairment, responsiveness to medication and electrode location in influencing the outcomes of DBS.^47^ Moreover, integrating patient-reported outcomes from real-world settings is essential to more fully assess the therapeutic effects and long-term satisfaction with treatment.^14,48,49^ By accounting for these variables, studies can better delineate the conditions under which DBS is most effective and identify subgroups of patients who may benefit from tailored interventions.

In conclusion, this study provides exploratory evidence that cortical oscillatory dynamics are associated with individual working memory outcomes in Parkinson’s disease patients undergoing STN-DBS. Baseline oscillatory power and task-related desynchronization may serve as candidate physiological markers for predicting working memory outcomes. This desynchronization may indicate a shift from a rigid neural state to a more adaptive network, potentially supporting cognitive performance. These physiological markers could also inform future studies on stimulation programming for more personalized treatment and serve as input signals for closed-loop systems that adjust stimulation in real-time to improve efficacy.

## Data Availability

Data are available upon request from the first author after institutional approval.

## Code availability

The GitHub repository https://github.com/neuromti/stn-dbs-working-memory-eeg includes the analysis scripts for this study.
